# Ultrasound-Assisted Extraction of Blackberry Seed Oil: Optimization and Oil Characterization

**DOI:** 10.3390/molecules28062486

**Published:** 2023-03-08

**Authors:** Petronela L. Matei, Iuliana Deleanu, Ana M. Brezoiu, Nicoleta A. Chira, Cristina Busuioc, Gabriela Isopencu, Mihaela Cîlțea-Udrescu, Elvira Alexandrescu, Anicuta Stoica-Guzun

**Affiliations:** 1 Department of Chemical and Biochemical Engineering, Faculty of Chemical Engineering and Biotechnologies, University “Politehnica” of Bucharest, 1-7 Polizu Street, 011061 Bucharest, Romania; 2 Department of Organic Chemistry “Costin Neniţescu”, Faculty of Chemical Engineering and Biotechnologies, University “Politehnica” of Bucharest, 1-7 Polizu Street, 011061 Bucharest, Romania; 3 Department of Science and Engineering of Oxide Materials and Nanomaterials, Faculty of Chemical Engineering and Biotechnologies, University “Politehnica” of Bucharest, 1-7 Polizu Street, 011061 Bucharest, Romania; 4 Department of Biotechnologies, Bioresources and Bioproducts for Bioeconomy, National Institute for Research and Development in Chemistry and Petrochemistry-ICECHIM, 202 Splaiul Independentei Street, 060021 Bucharest, Romania; 5 Department of Heterogeneous Systems, National Institute for Research and Development in Chemistry and Petrochemistry-ICECHIM, 202 Splaiul Independentei Street, 060021 Bucharest, Romania

**Keywords:** blackberry seed oil, ultrasound-assisted extraction, Box–Behnken design

## Abstract

Ultrasound-assisted extraction (UAE) was applied to extract oil from blackberry (BB) seeds. The effect of UAE conditions on oil recovery and quality was investigated. Favorable experimental conditions (ultrasound intensity (UI), extraction temperature, and time) were investigated using response surface methodology (RSM). A Box–Behnken design was used to predict optimized conditions for BB seed oil extraction. These conditions were as follows: 13.77 W/cm^2^ UI, 45 °C extraction temperature, and 15 min extraction time. The experimental value obtained for extraction efficiency under optimal conditions was 87 ± 0.34%, in good agreement with the optimized predicted value. UAE does not affect the oil composition and confers higher antioxidant values in BB seed oil in comparison with Soxhlet extraction.

## 1. Introduction

Fruit and vegetable processing is a source of wastes in the food industry that contain a large amount of bioactive compounds that could be valorized using new modern technologies [1,2,3,4,5]. The utilization of these wastes and of food industry by-products is an important part of a circular economy [6]. Thus, several objectives can be achieved: pollution reduction, obtaining of new raw materials for other industries and, obviously, economic efficiency increase [7]. Many fruits are raw materials for obtaining different foods: juice, jams, purée, marmalade, and other products. Typically, the juicing process generates a high quantity of waste known as pomace. The pomace obtained from different fruits is intensively studied as a source of valuable compounds, many of which have beneficial effects for human health [2]. For apple and orange pomace, biorefining schemes have already been developed [8,9,10,11,12].

Despite the high nutritional level of berries (wild or cultivated), their production level in Europe is low in comparison with oranges and apples. While an increase of berry consumption has been observed in recent years, this refers especially to fresh products. One of the problems associated with berries is that they are perishable, and for this reason, a part of the berry production must be industrially processed. It is estimated that only 40% of the berry production is consumed as fresh fruit. The large part that remains must be valorized to obtain different products, one of them being juice concentrate [13]. Thus, the pomace of berries could be obtained in large quantities, representing between 20–30% of the mass of the raw material [14]. Currently, these pomaces are used mostly as animal feed additive or soil fertilizer or are landfilled, generating high costs and environmental pollution [15]. Thus, important amounts of active substances and nutrients are lost.

Berry pomaces are constituted mainly of skin and seeds, with seeds being the main pomace fraction. Their high humidity and sugar content make them susceptible to microbial spoilage. Therefore, for further utilization, it is necessary to reduce the moisture content by drying them under certain conditions in order not to degrade thermolabile substances [16]. Approximately 68% of the dried pomaces is dietary fibers, followed by proteins and lipids. Berry pomace also contains high amounts of bioactive compounds with a high antioxidant effect, which is associated with health-promoting effects. For example, the water-soluble antioxidants are released in the juice, while those with low solubility remain in the pomace [16,17]. A simple way to valorize berry pomace is as flour for cookies and cakes, after drying and grinding [18,19]. More interesting are the biorefining schemes for all the berry wastes, not only pomace. Recently, Liu et al. (2021) proposed a sustainable blueberry waste recycling concept, which could be an example for the valorization of others berries as well [20]. Other contributions on this subject are also available [14,21].

In the large and diverse group of berries, blackberries have grown in popularity among consumers due to their health benefits and pleasant taste. Blackberries are rich not only in primary metabolites (such as sugars, proteins, and fats), but also in phytochemical compounds (the most important being phenolic acids), flavonoids, especially anthocyanins and flavanols (e.g., quercetin, kaempferol, and myricetin), and tannins (such as ellagitannins and proanthocyanidins), belonging to the class of phenolic compounds. Blackberry anthocyanins have been intensively analyzed, and the majority belong to cyanidin-based anthocyanins [22,23,24,25,26,27]. In terms of primary metabolites, BB have a low fat content (0.49%) and relatively higher sugars content (4.88–5.98%), with the most important sugars being glucose and fructose [22,27]. Fructose contents per 100 g of fresh fruits (FW) of the blackberry plant range from 1.38 to 3.34, and glucose contents range from 1.30 to 3.32 g/100 g FW [28]. Total phenolic and total anthocyanin values for BB fruits are very different based on variety, climate and soil conditions, stage of ripeness, and harvest and storage conditions. For example, total phenolic values in blackberries have been reported to range from 114 to 1056 mg/100 g FW [27], and total monomeric anthocyanin content has been reported to range from 14.7 to 124, expressed as mg cyanidin-3-glucoside equivalent/100 g FW [23].

All these compounds also have high antioxidant properties with beneficial effects on human health. Many minerals have been detected in BB fruits, the most important being potassium, calcium, magnesium, iron, zinc, and manganese [23]. Blackberries also contain organic acids as malic acid, isocitric acid, and citric acid as primary organic acids and trace amounts of other organic acids [27]. Blackberries are also a source of vitamins (such as C, K, E) and of dietary fibers. For example, vitamin C content is reported to range from 7.1 to 11.4 mg/100 g [26,28]. BB seeds also contain fatty acids (predominantly poly-unsaturated fatty acids) and other healthy compounds such as tocopherols, tocotrienols, sterols, ellagic acid, carotenoids, and phenolic compounds [23]. Not only BB fruits, but also aerial parts of the plant possess diverse valuable compounds with pharmacological effects [29,30].

As in the case of other berries, the BB pomace contains valuable phytochemicals with applications for food, pharmaceutical, and cosmetic products. Seeds, which represent the main part of the BB pomace, are a source of oil with a high value as raw material for the pharmaceutical and cosmetics industries, but also for the food industry. BB seed oil has a high content of polyunsaturated fatty acids (PUFA) with a favorable ratio between omega-6 and omega-3 fatty acids. Other valuable compounds of this oil are tocopherols, sterols, phenolic compounds, and carotenoids.

In all the pathways proposed for berry pomace biorefinery, the extraction of valuable compounds is an important step. Emerging extraction technologies are considered to be promising for the recovery of high added-value constituents from by-products of food industry or from agricultural wastes. Supercritical fluid extraction, pressurized liquid extraction, enzyme-assisted extraction, and ultrasound- or microwave-assisted extraction are intensively applied for the recovery of bioactive compounds from berry pomace [21,31,32,33,34]. Berry seeds are an important source of edible oil and have a high content of polyunsaturated fatty acids, tocopherols, tocotrienols, phytosterols, carotenoids, and phenolic compounds. Several emerging extraction technologies have been applied in order to enhance extraction yield and to obtain high-quality oil. The most important are supercritical fluid extraction, pressurized extraction, and ultrasonic-assisted extraction [35,36,37,38,39].

Ultrasound-assisted extraction (UAE) has some advantages that make it a very useful method for bioactive compound separation. These advantages include high efficiency, low extraction time, the possibility of extraction at low temperatures, low solvent consumption, and good extract quality [40]. However, some clarification is needed. Ultrasonic waves generated during ultrasonic-assisted extraction consist of compression and rarefaction cycles. Cavitation bubbles created during rarefaction collapse during compression and create extreme local conditions (high pressure and shear stress and high local temperature). The plant tissue is damaged (by erosion, pore formation, fragmentation, and other phenomena), and the active compounds can be released in the solvent, enhancing mass transport. The mechanism of UAE is more complicated, and the interaction between ultrasonic waves and plant material has been further investigated. If ultrasound intensity exceeds a high value, some bioactive compounds could be degraded, and the extraction yield decreases [40,41,42]. For this reason, the extract quality depends on the operating conditions.

Regardless of the chosen extraction method, one of the aims of research is to increase extraction yield. One way to achieve this goal is to optimize the extraction conditions, and many useful optimization methods are well-documented—one of them being response surface methodology (RSM). RSM fits data to a second-order polynomial model and has the advantage of a reduced number of experiments needed to estimate the interactions between the process variables. Many RSM designs are available, and for this study, we chose the Box–Behnken design (BBD) since it is more effective than other response surface designs [43,44].

The aim of this study was to optimize the process parameters of the ultrasound-assisted extraction of BB seed oil using RSM with a three-variable three-level BBD. A chemical characterization of the BB seed oil was also conducted to prove that the oil quality was not influenced by the ultrasound field. Special attention was paid to morphological changes of the plant material during ultrasound irradiation. To our knowledge, only one paper has presented data on the UAE of BB seed oil, but it neither contained an oil characterization nor highlighted plant-material morphological changes under ultrasound irradiation [45].

## 2. Results and Discussion

### 2.1. Morphological Characterization of BB Seeds after UAE

Preliminary experiments at different UI, but constant temperature (30 °C) and extraction time (10 min), were conducted with whole seeds to determine ultrasound (US) influence on plant material. The solid–liquid ratio was 1/20 (g/mL). For Soxhlet extraction, the same solid–liquid ratio was maintained, while the extraction time was 8 h.

**Figure 1:**
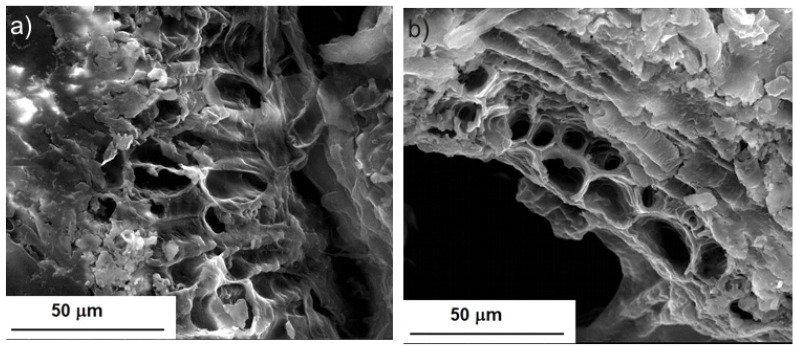
SEM images of whole BB seeds: (**a**) before extraction, and (**b**) after Soxhlet extraction.

**Figure 2:**
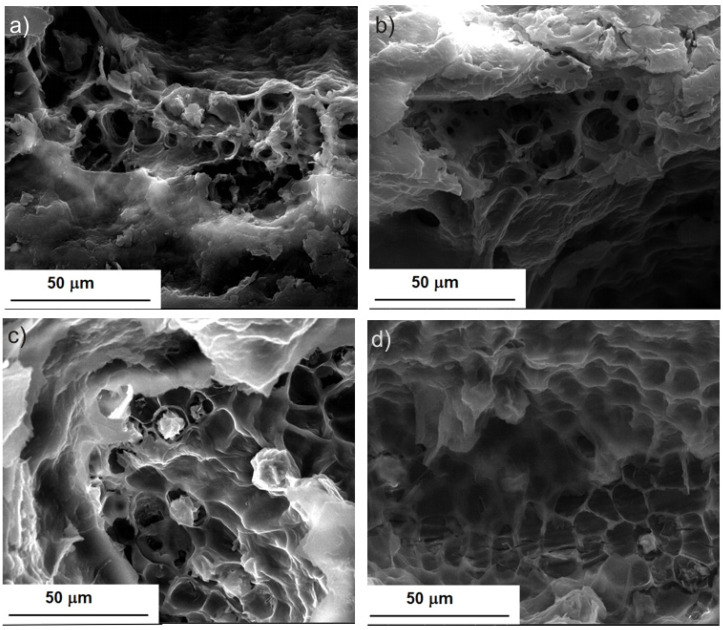
SEM images of the whole BB seeds after UAE at different UI: (**a**) UI = 5.06 W/cm^2^; (**b**) UI = 9.64 W/cm^2^; and (**c**,**d**) UI = 13.77 W/cm^2^.

Figure 1 shows the structure of the whole seed in the native state and after Soxhlet extraction. In Figure 1a, one can see the structure of the cell wall and the components of the plant tissue. After Soxhlet extraction, a depletion of some components of the seed tissue occurred, but the wall structure was not broken in comparison with the seed before extraction. In the seed images after UAE (Figure 2), the destructive nature of acoustic vibration is visible mainly at UI = 9. 64 W/cm^2^ (Figure 2b) by the appearance of fractures and openings in the plant wall. These changes could be attributed to the cavitation effect brought by the ultrasonic vibration. Even at UI = 5.06 W/cm^2^, a depletion of plant material is visible, and many holes and small craters were observed (Figure 2a). Compared to Soxhlet extraction, the material depletion was higher, especially at UI = 13.77 W/cm^2^ (Figure 2c,d), with the aspect being of an empty honeycomb.

### 2.2. Extraction Efficiency of UAE for BB Seed Oil Optimized by RSM

The UAE of BB seed oil was optimized using RSM. A BBD was used to predict optimized conditions. The experimental design matrix for the UAE of BB seed oil is presented in Table 1.

The second-order model obtained in terms of coded variables for extraction efficiency (*E*(%)) of BB seed oil using UAE is given by Equation (1):

(1) $$E\left(\%\right)=80.74+9.375X_{1}-2.495X_{1}^{2}+2.875X_{2}-3.495X_{2}^{2}+0.5X_{3}\;\;\;\;-2.745X_{3}^{2}-1.25X_{1}X_{2}-X_{1}X_{3}+X_{2}X_{3}$$

The ANOVA statistic for extraction efficiency (the response variable) proved that the regression model is highly significant, having a very high F-value of 46.67 and a low *p*-value of *p* < 0.0001. The lack-of-fit value of 7.25 and *p*-value of 0.394 imply that the lack of fit is not significant, which means that the model is adequate for the experimental data. The coefficient of variance (CV) also has a low value (CV = 1.77%) and provides better reproducibility. The quadratic model fitted well the experimental data if we consider the high values of R^2^ (0.983) and of Adj R^2^ (0.963). From the Adj R^2^ value, only 3.7% of the total variation could not be explained by the model.

Considering the *p*-value < 0.05 from Table 2 for each effect of the independent variables, the linear effect of UI, and the temperature and quadratic effects of UI, the temperature and extraction time are significant for extraction efficiency of BB seed oil. In these conditions, a simplified fitted quadratic model could be obtained by neglecting the non-significant terms (*p*-values > 0.05) in Equation (1). The new predictive quadratic model is expressed in Equation (2).

(2) $$E\left(\%\right)=80.74+9.375X_{1}-2.495X_{1}^{2}+2.875X_{2}-3.495X_{2}^{2}-2.745X_{3}^{2}$$

**Figure 3:**
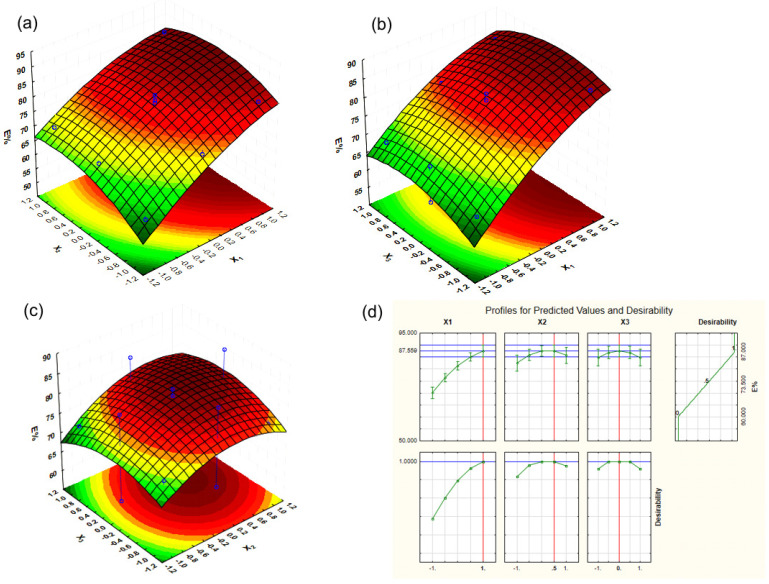
Response surface plot for interaction between the independent variables: (**a**) *X*_1_, *X*_2_; (**b**) *X*_1_, *X*_3_; (**c**) *X*_2_, *X*_3_; and (**d**) profiles for predicted values and desirability for BB seed oil extraction.

The extraction efficiency of BB seed oil under ultrasonic irradiation increases with increasing UI and extraction temperature (linear effect of variables *X*_1_ and *X*_2_). The response surface plots are a useful way to determine the effects of extraction parameters on the oil extraction efficiency. These plots were obtained by representing two variables within the experimental range and keeping the third variable constant at zero level. Figure 3a–c shows the effect of UI (*X*_1_), extraction temperature (*X*_2_), and extraction time (*X*_3_) on the BB seed oil extraction efficiency. It can be observed that the extraction efficiency increases with UI, which is in agreement with the fact that ultrasound intensity determines physical erosion and a high local stress, which increases the contact surface between solid and solvent and allows the fast release of the compounds in the solvent. Furthermore, increasing temperature increases the diffusion and solubility rate of the active compounds and enhances the mass-transfer rate. These influences are visible in Figure 3a. The influence of time is expressed through a quadratic term, and so the increase of extraction time is not favorable for obtaining a high degree of extraction efficiency (Figure 3c). In Figure 3b, one can observe that the influence of UI is more important than the influence of extraction time.

### 2.3. Desirability Optimization for BB Seed Oil

The extraction efficiency for UAE of BB seed oil was optimized by using the desirability function. The optimal conditions, expressed as coded variables, were found to be 1 for UI, 0.5 for extraction temperature, and 0 for extraction time. In the uncoded variables, this corresponds to 13.77 W/cm^2^ UI, 45 °C extraction temperature, and 15 min extraction time, where the optimum extraction efficiency for these conditions is 87.56%. Oil extraction efficiency profiles predicted by the model and the desirability level for the influencing factors for optimum oil extraction from BB seeds are presented in Figure 3d. The experimental value obtained for the optimal values of the independent variables was 87 ± 0.34%, in good agreement with the predicted value.

**Figure 4:**
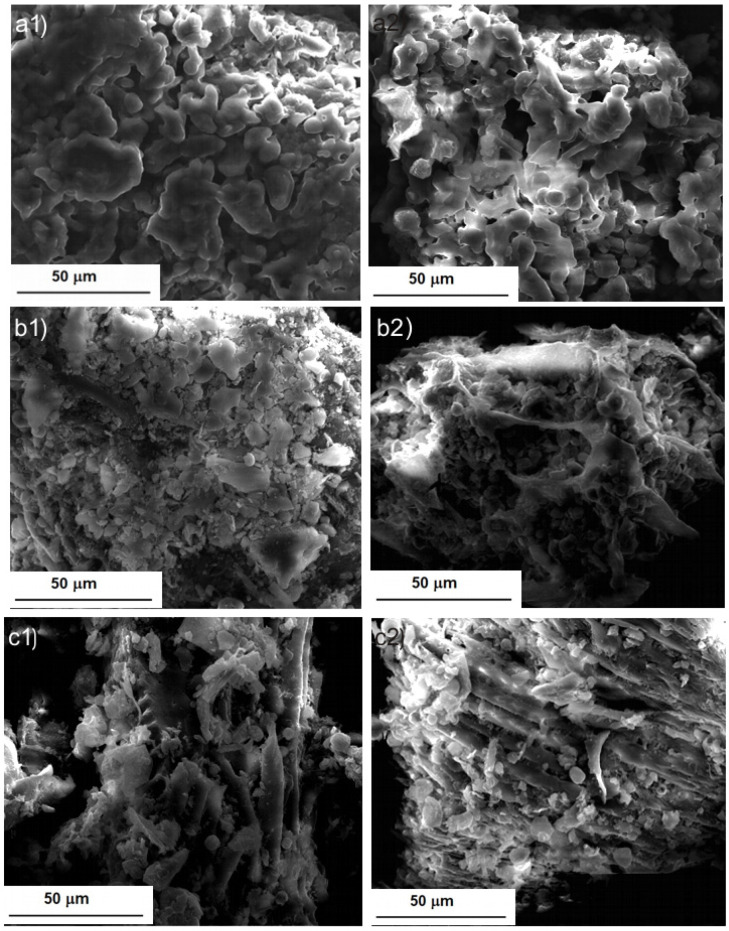
SEM images of BB seed powder: (**a1**,**a2**) before extraction; (**b1**,**b2**) after Soxhlet extraction; and (**c1**,**c2**) after Soxhlet extraction and extractive washing with methanol.

The ground BB seeds were analyzed using SEM before and after extraction using different techniques: Soxhlet extraction and UAE. In Figure 4(a1,a2), it can be seen that the initial powder is compact and has a relatively smooth surface, with some agglomerations of plant material. Soxhlet extraction led to the appearance of a rough seed powder surface with the appearance of voids, which evidence the dissolution of a part of the plant material in hexane, as can be observed in Figure 4(b1,b2). After extractive washing with a methanol solution, the powder had a fibrous structure, because a significative part of the soluble compounds (e.g., oil compounds (in hexane) and phenolic compounds (in methanol)) had left the plant tissue and were released in the used solvents. The images presented in Figure 4(c1,c2) support these assumptions.

**Figure 5:**
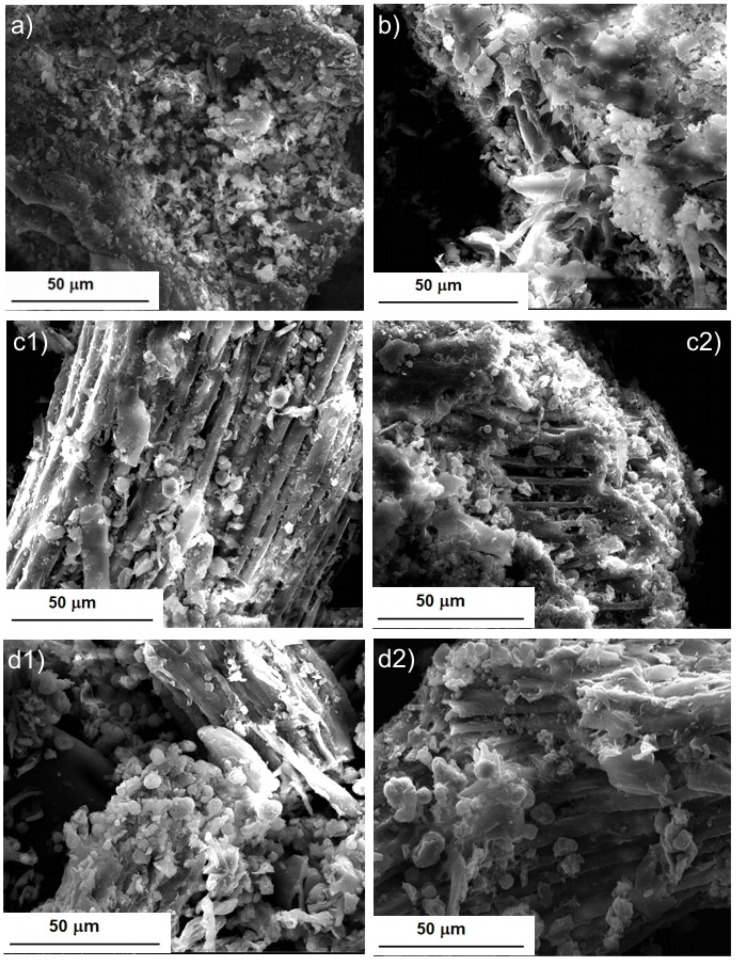
SEM images of BB seed powder after UAE: (**a**) UI = 5.06 W/cm^2^; (**b**) UI = 9.64 W/cm^2^; (**c1**,**c2**) UI = 13.77 W/cm^2^; and (**d1**,**d2**) UI = 13.77 W/cm^2^ and after the methanol extraction of defatted seed.

In Figure 5, SEM images of BB seed powder after UAE are presented. The extraction in the ultrasonic field brought dramatic changes in the structure of the plant material. At the lowest intensity of the ultrasonic field, the powder had a rough appearance, but with smaller solid particles compared to the powder obtained after Soxhlet extraction (Figure 5a compared to Figure 4(b1)). As UI increased (Figure 5b), the depletion of plant material could be observed. For the highest UI used, the fibrous structure of the seeds could be observed, because soluble compounds were released from the plant tissues as a result of the ultrasound action, especially due to the cavitation phenomenon (Figure 5(c1,c2)). The destructive action of the ultrasound waves on plant tissue is also visible in these images. The extractive washing with methanol also led to a reduction in the density of the plant material, in this case, because the polar compounds remaining after the extraction in hexane had dissolved in methanol. The images in Figure 5(d1,d2) prove these assumptions.

A certain similarity can be observed between Figure 4 and Figure 5, a similarity that is in favor of the use of the ultrasonic field, because what was observed after 8 hours of Soxhlet extraction (Figure 4(b1,b2)) was obtained after only 15 min of extraction assisted by ultrasound irradiation at the highest UI (Figure 5(c1,c2)). The images from Figure 5 are also in agreement with the results of the desirability function. The highest extraction efficiency was predicted for the highest UI (Figure 3d). Our observations agree with those reported by Khadhraoui et al. (2019), who studied various plant leaves under ultrasonic irradiation. The authors’ conclusion was that ultrasonic treatment induced structural changes of the leaf tissue [46].

### 2.4. BB Seed Oil Characterization

The BB seed oil content was determined to be 14.56 ± 0.53% (reported in the dried seeds, Soxhlet method). Generally, the berry seed oil content can be between 11-23% [47]. Our results are in agreement with those reported by Dimić et al. (2012), wherein the values for oil content were in the range of 13.97–14.37% as a function of drying conditions [48]. These comparisons are limited, because there are many variables that influence the lipid seed content, such as pedo-climatic conditions, temperature being one of the most important [23]. BB seed oil characterization is presented in Table 3.

As shown in Table 3, more than 82% of the BB seed oil consisted of unsaturated fatty acids, with the main fatty acid being linoleic acid (C18:2), accounting for approximately 65% of the total fatty acids. The oil also contained large amounts of oleic (C18:1, approx. 17%) and linolenic (C18:3, approx. 11%) acids, which explain the high iodine value of 157.6 g I_2_/100 g oil. The saturated fatty acids were represented by palmitic (C16:0) and stearic (C18:0) in ratios of 4.20% and 3.14%, respectively. All the identified fatty acids belong to the long-chain fatty acid class; the overall chain length of the constituent fatty acids is reflected in the saponification value of 191.5 mg KOH/g oil, which is a typical value for non-lauric vegetable fats [56]. The high amount of n-6 (linoleic acid) and n-3 (linolenic acid) identifies this blackberry seed oil as a valuable source of essential fatty acids. Compared to recently suggested alternate sources of essential fatty acids, such as blessed thistle (*Cnicus benedictus* L.) fruit oil, which contains similar levels of linoleic and oleic acids yet only traces of linolenic acid, blackberry seed oil shows significantly larger amounts of C18:3, consequently showing a balanced profile of the n-6/n-3 fatty acids [57]. On the other hand, other recently reported alternate sources of essential fatty acids, such as common purslane (*Portulaca oleracea* L.) seeds or sea buckthorn (*Hippophaë rhamnoides* L.) seeds, contain more than 25% linolenic acid [58,59].

The positive health effects of blackberry seed oil are implied by its very low atherogenic and thrombogenic indices (0.04 and 0.10, respectively), as well as by its high values of desaturase and health-promoting indices (81.38 and 29.51, respectively). In addition, the prevalence of poly-unsaturated fatty acids provides BB seed oil with hypocholesterolemic attributes; this hypothesis is confirmed by the high value of the H/H ratio of 29.51, as shown in Table 3. For comparison, dairy fats (cow, sheep, goat, ewe), which are generally recognized for their hypercholesterolemia effect that is attributed to their fatty acid profile rich in saturated and mono-unsaturated fatty acids, have HH indices ranging between 0.32–1.29 [60]. It is worth noting that trans fatty acids were not detected in these BB seed oils, neither in the conventionally extracted samples (Soxhlet) nor those extracted with UAE.

**Figure 6:**
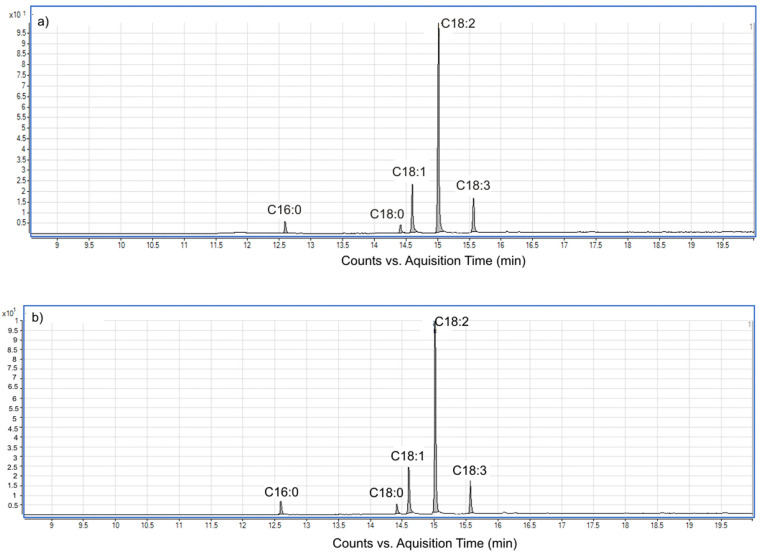
Gas chromatogram of BB seed oil: (**a**) Soxhlet extraction and (**b**) UAE.

Consequently, blackberry seed oil appears to be an attractive source of beneficial fatty acids, and it may therefore constitute an important ingredient for supplementation to obtain value-added food products. Chromatograms of BB seed oil obtained via Soxhlet extraction and UAE in *n*-hexane are presented in Figure 6. The fatty acid profile of BB seed oil is similar regardless of the extraction technique, as can be seen in Table 3 and Figure 6.

**Figure 7:**
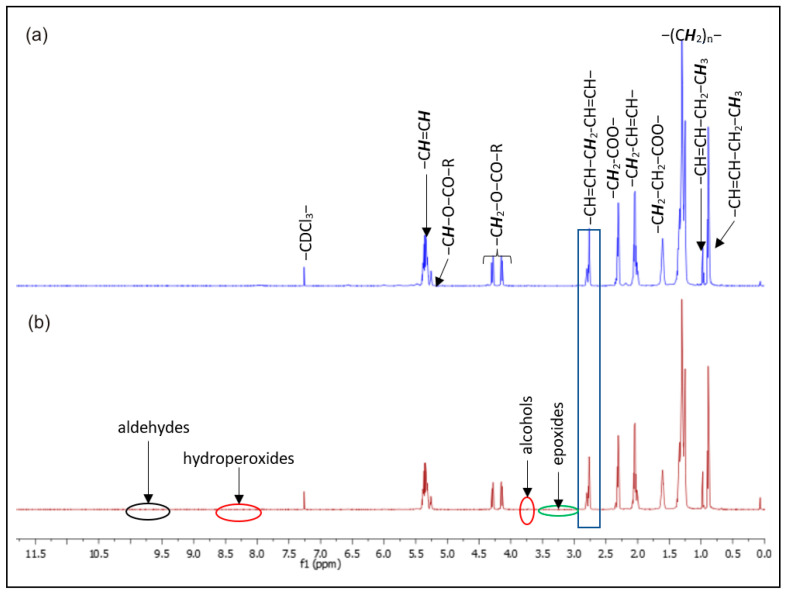
^1^H-NMR spectra of BB seed oil obtained by (**a**) Soxhlet extraction and (**b**) UAE.

The occurrence of oxidative processes during the ultrasound treatment was investigated via proton nuclear magnetic resonance (^1^H-NMR) spectroscopy. Figure 7 shows the stacked spectra of BB seed oil obtained by Soxhlet and UAE, respectively. The assignment of the oil resonances is presented in Table 4.

As shown in Figure 7, there are no significant differences between the two oil samples, as they both display the same signals, ascribable to the terminal methyl protons (0.85 ppm and 0.95 ppm in the case of the linolenic acyl moiety), methylene groups situated in the β position relative to the ester groups (1.64 ppm), allylic protons (2.02 ppm), methylene groups adjacent to the ester groups (2.26 ppm), bis-allylic protons (2.76 ppm) from the linolenic and linoleic acyl chains, the four protons from the sn-1/3 positions of the glycerol backbone (4.19 ppm), and the corresponding sn-2 proton (5.20 ppm). The most shielded resonance at 5.29 ppm is assigned to the unsaturated protons (–CH=CH–) from all the unsaturated fatty acyl chains, while the rest of the methylene groups resonate at around 1.24 ppm, contributing to the most prominent signal. All the spectral data confirm the fatty acid profile of the BB seed oil, determined chromatographically. In addition, upon examining the regions at 9.41–10.10 ppm (corresponding to protons from aldehyde groups), 8.00–8.50 ppm (corresponding to the resonances from the hydroperoxide functions), 3.85–3.89 ppm (alcohols, resulted from hydroperoxides breakdown), and 2.40–3.60 ppm (assigned to the protons from the oxirane rings of epoxides) [62], the ^1^H-NMR spectra clearly show that no primary (hydroperoxides) or secondary (such as epoxy fatty acids, alcohols, and aldehydes) oxidation products exist in the BB seed oils (neither obtained by Soxhlet nor by UAE) in detectable amounts, indicating that the ultrasound treatment had not initiated peroxidation processes. The absence of significant oxidative processes in both the cases of Soxhlet- and UAE-obtained BB seed oil was confirmed by the low peroxide values of 4.23 and 4.31 meq active oxygen/kg oil, respectively (Table 3).

Hence, although BB seed oil is highly unsaturated (IV = 157.6 g I_2_/100 g oil), and thus prone to undergo peroxidation at the bis-allylic positions from linolenic and linoleic acyl moieties, the absence of any oxidation marks may be due to its intrinsic protection conferred by the polyphenols from the berries’ skin [63], as in the case of other seed oils [57]. Consequently, the ultrasound treatment at the optimal extraction conditions (i.e., UI = 13.77 W/cm^2^, extraction temperature = 45 °C, and extraction time = 15 min) does not alter the characteristic fatty acid profile of BB seed oil and does not initiate oxidative processes.

### 2.5. UV–Vis Spectra of BB Seed Oil

**Figure 8:**
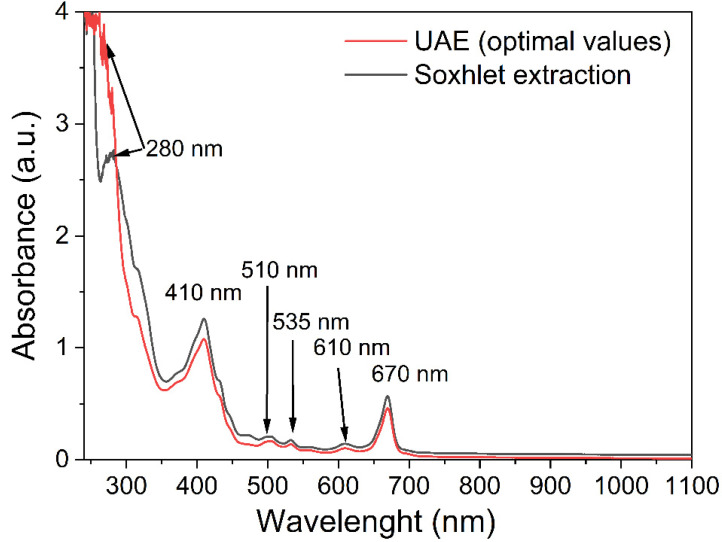
UV–Vis spectra of BB seed oil obtained by Soxhlet extraction and UAE.

The UV–Vis spectra of BB seed oil obtained by Soxhlet extraction and UAE are presented in Figure 8. Usually, the absorption from 280 nm is associated with the formation of trienes during the lipid oxidation and sometimes is correlated with the peak from 232 nm being due to dienes formation [47]. Other authors considered that this peak (at about 280 nm) is associated with tocopherols and fatty acids (palmitic, oleic, linoleic, and stearic acid) [64]. Peaks from 510 nm and 535 nm could be associated with the anthocyanin amount in correlation with a good amount of TAC (Table 5), while the peaks from 610 nm and 670 nm are due to pheophytin and chlorophyll content, respectively [65].

### 2.6. Total Phenolic Content (TPC) and Anthocyanin Content (TAC) of BB Seed Oil and Defatted Seed Methanolic Extracts and Antioxidant Activity

The total phenolic content (TPC) of BB seed oil or defatted seed flour methanolic extracts was determined based on the Folin–Ciocalteu method; the obtained results are expressed as mg gallic or caffeic acid equivalents per 100 g of dried seed flour (Table 5). For total anthocyanin pigments, the pH-differential determination was applied using the extinction coefficient of cyanidin-3-glucoside. A good correlation between the TPC and TAC was observed, emphasizing that ultrasound-assisted extraction increased the amount of phytocompounds recovered for both BB seed oil and defatted seed flour extract with significantly larger amounts in the latter, which could be associated with a higher solubility of polyphenols in polar solvents [66]. The amount of polyphenols present in BB seed oil ranged from 3.19–3.24 mg GAE/100 g dw (21.92–26.34 mg GAE/100 g oil) with an amount of anthocyanin of 0.41–0.46 mg C3GE/100 g dw. These values are lower than those reported by Correa et al. (2021) for an n-hexane BB oil (75.26 ± 1.75 mg GAE/100 g oil) [38]. However, an improved polyphenolic content (1432.82–1721.65 μmol GAE/L oil) was observed in the BB seed oil extract as compared with the values reported by Luo et al. (2021) of 52.72 μmol GAE/L oil, which could be associated with their use of a different extraction solvent, an acetone solution [67].

The polyphenol extract from defatted BB seed flour showed good amounts of polyphenols (29.84–34.71 mg GAE/g dw) and colored anthocyanin pigments (37.21–40.86 mg C3GE/100 g dw). Considering that the pomace also encompasses significant amounts of pulp and skins associated with higher levels of polyphenols, especially anthocyanins [68], these values are similar to those reported by Jazic et al. (2018) for four different pomaces from different cultivars by Soxhlet extraction in 80% ethanol (26.30–50.16 mg GAE/g pomace). Moreover, Sariburun et al. (2010) reported values for TPC (1787.3–2062.3 mg GAE/100 g fresh weight) and TAC (12.4–24.8 C3GE/100 g fresh weight) that were lower than those presented in this study, probably due to the starting material humidity [69].

The antioxidant capacity of the methanolic extract obtained from BB seed oil, presented in Table 5, is lower compared to the methanolic extract of the defatted BB seed residue. These results are in agreement with the results obtained for TPC and TAC.

## 3. Materials and Methods

### 3.1. Materials

All reagents used were of analytical grade, and all solutions were prepared using deionized water. The following reagents were purchased from Sigma-Aldrich Chemie GmbH, Taufkirchen (München, Germany) and were used without further purification: sodium carbonate (Na_2_CO_3_) (CAS No: 5968-11-6), n-hexane (CAS No: 110-54-3), Folin Ciocalteu reagent (CAS No: 110-54-3), methanol (CAS No: 67-56-1), gallic acid (CAS No. 149-91-7), caffeic acid (CAS No: 501-16-6), 2,2-Diphenyl-1-picrylhydrazyl (DPPH) (CAS No:1898-66-4), phosphate-buffered saline (CAS No: 806552), (S)-Trolox methyl ether (CAS No: 135806-59-6). Buffer solutions of pH 1 and 4.6 were purchased from Honeywell Fluka, Buchs (St. Gallen, Switerzerland) and used as received without further purification.

### 3.2. Vegetal Material

Blackberries of a wild variety were collected in Prahova County (Măneciu, Romania) in July-August 2022 and used as vegetal material. The fruits were manually cleaned, washed, and then stored at 4 ± 1 °C until their processing. BB juice was extracted by cold-pressing using a Kuvings-Slow juicer (NUC Electronics Co., Ltd., Daegu, Taegu-jikhalsi, South Korea). A pomace consisting of seeds, peels, and pulp fraction was obtained as by-product. The pomace was dried at 50 °C in a food dehydrator (Tribest Sedona Express SDE-P6280, Anaheim, CA, USA). After drying, the seeds were manually separated from the dried pomace and then stored at 4 ± 1 °C in sealed plastic bags before oil extraction. The final moisture content of the seeds was determined at 120 °C. Then, BB seeds were sufficiently ground using a common coffee mill to obtain a very fine powder. The remaining coarse particles were removed using a vibratory sieve shaker, and the fraction with a particle diameter of lower than 0.2 mm was used for all the experiments.

### 3.3. Extraction Techniques

#### 3.3.1. Soxhlet Extraction

The initial oil content in BB seeds was determined using a laboratory Soxhlet extractor and *n*-hexane as a solvent. About 10 g of grinded seeds were weighted and subjected to continuous (Soxhlet) extraction with 200 mL of *n*-hexane for 8 h. The extraction was performed in triplicate. The moisture content of the seeds (5.5 ± 0.2%) was determined using a thermo-balance OHAUS MB23. The oil extraction yield (*Y*_oil_ (%)) was calculated with Formula (3):

(3) $$Y_{oil}\left(\%\right)=\frac{M_{oil}}{M_{ds}}\times 100$$

where *M_oil_* is the mass of extracted oil (g), and *M_ds_* is the mass of the dried seed (g).

#### 3.3.2. Ultrasound Assisted Extraction (UAE)

UAE was carried out using a 500 W Ultrasonic Processors–VCX Series (Sonics & Materials, Inc., Newtown, CT, USA). Samples of dried and grinded BB seeds were placed in a glass vessel close-fitting the US probe top diameter, equipped with a reflux condenser, and operated at different operating parameters (e.g., temperature, extraction time, and UI) according to the BBD. The used solvent was n-hexane, and the solid–liquid ratio was 1/20 (g/mL).

### 3.4. Experimental Design and Statistical Analysis

Response surface methodology (RSM) using Box–Behnken design (BBD) was chosen to determine the optimal extraction conditions for BB seed oil using the STATISTICA 10 software package (Stat Soft Inc., Tulsa, OK, USA). The Box–Behnken design is often used to analyze the effect of the independent variables to the response. Despite some disadvantages, it is considered more efficient than other response surface designs and has the advantage of requiring fewer experiments.

Based on the preliminary experiments, ultrasonic intensity (UI), extraction temperature, and extraction time were considered as the main important variables.

Ultrasonic intensity was calculated by Equation (4):

(4) UI=4PπD2

where *UI* is the ultrasonic intensity (W/cm^2^), P is the ultrasound power (W) measured using calorimetric method, and *D* is the internal diameter (cm) at the tip of the probe [70].

Table 6 presents the uncoded and coded levels of the three independent factors, designated as *X*_1_ (UI, ultrasonic intensity), *X*_2_ (extraction temperature), and *X*_3_ (extraction time). The solid–liquid ratio was maintained constant at 1:20 g/mL in all UAE experiments. The values of UI correspond to 20, 30, and 40% ultrasound amplitude.

The response variable for UAE was the extraction efficiency, calculated using Equation (5), where *M_oil_* is the mass of the extracted oil (g), *M_ds_* is the mass of the dried seeds (g), and *S_oil_* is the oil content determined by Soxhlet extraction (g oil/g dried seeds):

(5) $$E\left(\%\right)=\frac{M_{oil}}{M_{ds}\times S_{oil}}\times 100$$

A second-order polynomial regression model, given by Equation (6), was used to study the effect of the independent variable upon extraction efficiency:

(6) $$Y=\beta_{0}+\Sigma \beta_{i}x_{i}+\Sigma \beta_{ii}x_{i}^{2}+\Sigma \beta_{ij}x_{i}x_{j}$$

where *Y* is the response variable, *β*_0_ is the constant term, *β_i_* are the linear coefficients, and *β_ii_* and *β_ij_* are quadratic and interaction coefficients, respectively.

The statistical significance of the regression model was tested by analysis of variance (ANOVA) and by Fisher’s (F-test) and *p*-value. The interactions between variables were represented using three-dimensional response surface plots. In this study, the response was optimized by a desirability function using the STATISTICA 10 software package (Stat Soft Inc., Tulsa, OK, USA).

### 3.5. Analysis of Fatty Acid Composition of BB Seed Oil and Determination of the Oil Quality Indices

Fatty-acid methyl esters (FAME) were prepared from BB seed oil via transesterification with methanol, under alkaline conditions. The method is generally described elsewhere [57,59]. Briefly, 100 µL oil was pipetted into a screw-capped vial, and then 2 mL of n-hexane and 0.2 mL of a KOH methanolic solution (2 N) were added and vigorously shaken. After heating at 60 °C for 5 min, the mixture was allowed to stand until cooled to room temperature and diluted with an additional 1 mL of n-hexane. An aliquot of 150 µL was taken from the upper layer and further separated through GC. An Agilent 7890B GC (Agilent Technologies, Santa Clara, CA, USA) GC system equipped with an auto-sampler, 5975 C VL MSD triple axis MS detector, and a Supelco SPTM 2560 capillary column (100 m length, 0.25 mm inner diameter, and 0.2 μm film thickness) were used to determine the concentration of fatty acids in the CB oil. Helium (1.0 mL/min) was used as a carrier gas; the split ratio was 1:100. The oven temperature was set as follows: 90 °C (1 min), 90–240 °C (10 °C/min), and 240 °C (4 min). FAME solutions (150 μL) were injected into the column, and the fatty acids were identified by comparing the retention times corresponding to the peaks with those of a standard mixture (Supelco^®^ 37 Component FAME Mix, purchased from Sigma-Aldrich Chemie GmbH (Taufkirchen, Germany)). The fatty acid profile of the BB seed oil was determined from the peak areas adjusted by the response factors of the detector; these factors were calculated for each FAME in the standard mixture by reporting the unit area of each peak to the unit area of oleic acid methyl ester peak, taken as a reference. The response factors of the detector were calculated as an average of five determinations.

Oil health indices and oil technical quality indices were computed based on the fatty acid composition data using formulas available in the literature. These indices are AI = atherogenic index [49]; TI = thrombogenic index [49]; P/S ratio = poly-unsaturated/saturated fatty acids ratio; H/H ratio = hypocholesterolemic/hypercholesterolemic fatty acids ratio [50]; HPI = health-promoting index [51]; DI = desaturase index [52]; IV = iodine value [53]; SV = saponification value [54]; and PV = peroxide value [55]. PV was determined by iodometric titration, based on the fact that the iodide ions from the KI are stoichiometrically oxidized to molecular I_2_ by hydroperoxides, which can subsequently be titrated with a standard Na_2_S_2_O_3_ solution in the presence of starch as an indicator [55].

### 3.6. ^1^H-NMR Analysis

The NMR spectra were recorded on a Bruker NMR 600 MHz Advance (Bruker, Rheinstetten, Germany)—corresponding to the resonance frequency of 600.12 MHz for the ^1^H nucleus—equipped with a 5 mm multinuclear indirect-detection z-gradient probe head. Samples were dissolved in CDCl_3_ and transferred in 5 mm NMR tubes. The NMR spectra were recorded using a standard pulse sequence, as provided by Bruker, with TopSpin 3.5.pl6 spectrometer software. The ^1^H-NMR spectrum was recorded with 16 scans and a delay time of 1s. The CDCl_3_ (solvent) resonance was calibrated at 7.26 ppm.

### 3.7. UV–Vis Spectra

UV–Vis spectra of the BB seed oil were recorded using a Shimadzu UV-1800 (Shimadzu Corporation, Kyoto, Japan).

### 3.8. Determination of Total Phenolic Content (TPC), Total Anthocyanin Content (TAC), and Antioxidant Capacity of BB Seed Oil and of Defatted BB Seed Methanolic Extract

#### 3.8.1. Sample Preparation

TPC and TAC content was determined for the methanolic extract of the BB seed oil and of the defatted seed flour that was obtained as residue after extraction. The methanolic extract of the BB seed oil was obtained using a procedure proposed by Thilakarathna et. al. (2023) [71]. Defatted seed flour obtained through Soxhlet extraction and UAE was extracted for 16 h at room temperature in an aqueous methanol solution (60% methanol *v*/*v*) with a solid–liquid ratio of 1/10. After extraction, the samples were centrifuged, and the methanolic extract was analyzed. Extractions were performed in triplicate.

#### 3.8.2. Total Phenolic Content (TPC) and Total Anthocyanin Content (TAC)

The total polyphenolic content was computed based on a standard curve for either gallic or caffeic acid (in 50–450 μg/mL range) by obtaining a blue-molybdenum complex using a polyphenol ethanolic solution with diluted Folin–Ciocalteu reagent (1/10 reagent–water ratio) in basic medium after 1.5 h incubation in dark static conditions. This method is described elsewhere [72]. The solution absorbance values were afterwards measured at both 765 nm (y = 0.00945x + 0.017; R^2^ = 0.9995 for gallic acid, y = 0.01054x + 0.017; R^2^ = 0.9999 for caffeic acid) and 650 nm (y = 0.00965x + 0.017; R^2^ = 0.9998 for gallic acid, y = 0.01048x + 0.017; R^2^ = 0.9997 for caffeic acid) wavelengths, and the obtained values were presented as an average of four determinations.

The content of total anthocyanin pigments was determined based on the procedure described by Lee et al. (2005) [73] with slight modifications presented by Brezoiu et al. (2020) [74] that emphasize the differences in solution absorbance of anthocyanin pigments at pH 1 in oxonium form (highest absorption) and the colorless one at pH 4.5 (lowest absorption). Therefore, extracts were properly diluted with the buffer solution of pH 1, and the dilution factor was determined. Using the same dilution factor, the extract was then diluted with the buffer solution of pH 4.5, and the solution absorbance was measured at 520 nm and 900 nm towards the corresponding buffer solution. Total anthocyanin content, expressed as cyanidin-3-glucoside equivalent, was determined using Equation (7):

(7) $$TAC\left(mg\;C3GE/L\right)=\frac{A\times M\times f}{\varepsilon \times l}\times 1000$$

where *A* = (*A*_520nm_ − *A*_900nm_)_pH 1_ − (*A*_520nm_ − *A*_900nm_)_pH 4.6_, *M* is the molecular mass of cyanidin-3-gluoside(C3G) at 449.2 (g/mol), *f* is the dilution factor, *l* is the pathway length (cm), *ε* is the extinction coefficient for C3G at 26,900 L·mol^−1^·cm^−1^, and 1000 is the conversion factor from g to mg.

#### 3.8.3. Antioxidant Capacity

The DPPH (2,2-diphenyl-1-picryl-hydrazyl-hydrate) free-radical method is an antioxidant assay based on electron transfer that produces a violet solution in solvents such as ethanol or methanol. This free radical, which is stable at room temperature, is reduced in the presence of an antioxidant molecule, causing the discoloration of the alcoholic solution. The antioxidant activity of the samples was determined using the DPPH antioxidant assay, as reported by Akter et al. (2019), with appropriate modification [75]. The tests were carried out in triplicate. A volume of 400 microliters of methanolic (60% *v*/*v* methanol/water) extracts was mixed with the methanolic solution of DPPH, vortexed (Stuart Scientific SA8 vortex mixer, UK) for 10 sec, and left in the dark at room temperature for 30 min. The absorbance was read at 517 nm with a UniSpec2 Spectrophotometer (LLG-Labware, Meckenheim, Germany). The TEAC-equivalent concentration (μg/g probe) was determined from a calibration curve, and the antioxidant efficiency (*AE*) of the samples compared to a control sample was calculated with Equation (8):

(8) $$AE,\%=\frac{Abs_{0}-Abs_{p}\;}{Abs_{0}}\times 100$$

where *Abs*_0_ is the absorbance of the blank, and *Ab_sp_* is the absorbance in the presence of the samples.

### 3.9. BB Seed Characterization

The structure of BB seeds and of the BB seed powder before and after solvent extraction was visualized with a FEI Quanta Inspect F Scanning Electron Microscope (SEM). All samples were gold-coated prior to SEM examination.

## 4. Conclusions

UAE was used to extract BB seed oil, and the extraction process was optimized using a statistical method based on the response surface methodology in order to identify and quantify the variables that may maximize the relative extraction efficiency. The optimized values for UAE were 13.77 W/cm^2^ UI, 45 °C extraction temperature, and 15 min extraction time. Under these operation parameters, the extraction efficiency value was 87 ± 0.34%. These conditions could be appreciated as mild, which prevents oil degradation. The oil analyses revealed that the fatty acid profile of BB seed oil is similar regardless of the extraction technique (UAE or Soxhlet). Antioxidant capacity was enhanced by UAE both for BB seed oil extract and for the defatted seeds extract. These results indicate that UAE is an efficient method for obtaining BB seed oil due to its high efficiency and the high quality of the extracted oil. Our study opens the possibility of integrating the UAE of BB seed oil into a biorefining scheme of BB pomace. BB waste consists of seeds and peels and, as has already been shown, contains a high amount of bioactive substances. Extraction could be used for the recovery of these valuable substances, namely, oils and antioxidant compounds, but for industrial use, it is necessary to optimize the extraction conditions. The conventional extraction techniques are time-consuming, and this can limit their applicability in the industrial sector. However, new extraction methods, UAE being one of them, could overcome this limitation. In our own preliminary experiments, the extraction efficiency was higher after 15 min for BB seed oil extraction using UAE compared with two hours of batch extraction, and there are references that prove that UAE is scalable for industrial applications [76]. While one of the problems associated with UAE is the possibility of degradation of some of the extracted active compounds, if the extraction conditions are optimized so that the quality of the final extract is maintained, then UAE becomes a promising alternative to classic extraction methods.

## Figures and Tables

**Table 1:** BBD matrix of independent variables and the corresponding experimental and predicted values for BB seed oil extraction efficiency.

Run	*X*_1_	*X*_2_	*X*_3_	*E_exp_* (%)	*E_predicted_* (%)
1	−1	−1	0	60 ± 0.17	61.25
2	−1	0	−1	65 ± 0.25	64.62
3	−1	0	1	68 ± 0.13	67.62
4	−1	1	0	70 ± 0.09	69.50
5	0	−1	−1	73 ± 0.45	72.12
6	0	−1	1	72 ± 0.34	71.12
7	0	0	0	81 ± 0.25	80.74
8	0	1	−1	75 ± 0.16	75.87
9	0	1	1	78 ± 0.34	78.87
10	1	−1	0	82 ± 0.53	82.50
11	1	0	−1	85 ± 0.17	85.37
12	1	0	1	84 ± 0.40	84.37
13	1	1	0	87 ± 0.32	85.75
14	0	0	0	79 ± 0.19	80.74
15	0	0	0	82.7 ± 0.25	80.74
16	0	0	0	81 ± 0.16	80.74
17	0	0	0	80 ± 0.34	80.74

**Table 2:** Analysis of variance (ANOVA) for the response surface model of the independent variables for UAE of BB seed oil.

Term	Sum of Squares	DF	Mean Square	*F*-Value	*p*-Value
Model	907.3533	9	108.8170	47.67729	0.000019 ^a^
*X*_1_	703.1250	1	703.1250	372.4179	0.000042 ^a^
*X*_2_	66.1250	1	66.1250	35.0238	0.004083 ^a^
*X*_3_	2.0000	1	2.0000	1.0593	0.361533
*X*_1_^2^	26.2106	1	26.2106	13.8827	0.020368 ^a^
*X*_2_^2^	51.4317	1	51.4317	27.2414	0.006430 ^a^
*X*_3_^2^	31.7264	1	31.7264	16.8042	0.014862 ^a^
*X*_1_ *X*_2_	6.2500	1	6.2500	3.3104	0.142969
*X*_1_ *X*_3_	4.0000	1	4.0000	2.1186	0.219218
*X*_2_ *X*_3_	4.0000	1	4.0000	2.1186	0.219218
Lack of Fit	7.2500	3	2.4167	1.2800	0.394875
Pure error	7.5520	4	1.8880		
Total SS	922.1553	16			
*R*^2^ = 0.983	Adj *R*^2^ = 0.963		CV = 1.77%		

^a^ The values are significant at *p* < 0.05.

**Table 3:** BB seed oil characterization as obtained by Soxhlet extraction and UAE.

No.	Fatty Acid	Lipid Number	Fatty Acid Profile of BB Seed Oil (Molar) *	
			Soxhlet Extraction (*n*-hexane, 8 h)	UAE (13.77 W/cm^2^ UI, 45 °C, 15 min) (Optimum Values)
1	Palmitic	C16:0	4.20 ± 0.16	4.75 ± 0.18
2	Stearic	C18:0	3.14 ± 0.02	3.55 ± 0.09
3	Oleic	C18:1	16.52 ± 0.57	17.21 ± 0.63
4	Linoleic	C18:2	64.86 ± 0.96	63.53 ± 0.78
5	α-Linolenic	C18:3	11.28 ± 0.34	10.97 ± 0.36
∑ SAFA			7.34	8.3
∑MUFA			16.52	17.21
∑PUFA			76.14	74.5
n-6/n-3			5.73	5.79
Total fat (g/100 g dried seeds)			14.56 ± 0.53	
**Oil health indices** **				
AI			0.04	0.04
TI			0.10	0.11
P/S ratio			10.37	8.98
H/H ratio			29.51	25.83
HPI			29.51	25.83
DI			81.38	80.74
**Oil technical quality indices**				
	IV (g I_2_/100 g oil) **		157.6 ± 0.8	157.8 ± 0.9
	SV (mg KOH/g oil) **		191.5 ± 0.5	191.6 ± 0.9
	PV (meq. active oxygen/kg of oil)		4.23 ± 0.34	4.31 ± 0.34

* Reported as mean values (three replicates) ± sd. ** Computed based on the fatty acid composition data. AI = atherogenic index [49]; TI = thrombogenic index [49]; P/S ratio = poly-unsaturated/saturated fatty acids ratio; H/H ratio = hypocholesterolemic/hypercholesterolemic fatty acids ratio [50]; HPI = health-promoting index [51]; DI = desaturase index [52]. IV = iodine value [53]; SV = saponification value [54]; PV = peroxide value [55]. SAFA: saturated fatty acids. MUFA: monounsaturated fatty acids. PUFA: polyunsaturated fatty acids. n-6/n-3 is the ratio of n-6 to n-3 fatty acids.

**Table 4:** Chemical shifts and assignment of 1H-NMR resonances of BB seed oil [61].

No.	δ (ppm)	Proton	Compound
**1**	0.85	–CH_2_–CH_2_–CH_2_–C***H***_3_	all acids except linolenic acid
**2**	0.85	–CH=CH–CH2-C***H***_3_	linolenic acid
**3**	1.24	–(C***H***_2_)_n_-	all fatty acids
**4**	1.64	–C***H***_2_–CH_2_–COO–	all fatty acids
**5**	2.02	–C***H***_2_–CH=CH–	allylic acids (from all unsaturated fatty acids)
**6**	2.26	–C***H***_2_–COO–	all fatty acids
**7**	2.76	–CH = CH–C***H***_2_–CH=CH–	*bis*-allylic protons (from linolenic and linoleic acid)
**8**	3.60	–COO–C***H***_3_	methyl ester moiety
**9**	4.19	–C***H***_2_OCOR	H in the *sn*-1/3 position of the glycerol backbone
**10**	5.20	–C***H***OCOR	H in the *sn*-2 position of the glycerol backbone
**11**	5.29	–C*H*=C***H***-	all unsaturated fatty acids

**Table 5:** TPC, TCA, and antioxidant activity of BB seed oil methanolic extract and of defatted seed methanolic extract.

Methanolic Extract	TPC		TAC	TEAC	AE
	mg GAE/100 g dw	mg CAE/100 g dw	mg C3GE/100 g dw	(μg/g Extract)	%
BB seed oil (Soxhlet extraction)	3.19 ± 0.17	2.90 ± 0.15	0.41 ± 0.02	72.91 ± 0.35	33.11
BB seed oil (UAE)	3.34 ± 0.19	3.48 ± 0.20	0.46 ± 0.02	81.73 ± 0.6	28.51
Defatted BB seed residue (Soxhlet extraction)	2984.71 ± 88.28	2712.69 ± 80.23	37.21 ± 1.19	266.71 ± 7.27	89.07
Defatted BB seed residue (UAE)	3471.12 ± 73.73	3155.04 ± 66.98	40.86 ± 2.51	267.50 ± 9.25	89.37

**Table 6:** Independent variables with uncoded and coded levels for UAE of BB seed oil.

Independent Variables	Symbol	Coded Levels		
		−1	0	1
UI (W/cm^2^)	*X*_1_	5.06	9.64	13.77
Extraction temperature (°C)	*X*_2_	30	40	50
Extraction time (min)	*X*_3_	10	15	20

## Data Availability

The data presented in this work are available in the article.
